# Tobacco Quitlines May Help Exclusive Vapers Quit: An Analysis of Data From an Employer-Sponsored Quitline

**DOI:** 10.5888/pcd20.220300

**Published:** 2023-06-08

**Authors:** Etta Short, Kelly M. Carpenter, Kristina Mullis, Chelsea Nash, Katrina A. Vickerman

**Affiliations:** 1 Optum, Eden Prairie, Minnesota

## Abstract

Numerous studies have supported the effectiveness and cost-effectiveness of quitlines for cigarette smoking cessation, but how effective they are for vaping cessation has not been established. Our secondary analysis examined quitline data on participants in employer-sponsored quitlines in the US run by Optum, Inc to compare quit rates among callers who were exclusive vapers (n = 1,194) with those who were exclusive smokers (n = 22,845). We examined data from the time of quitline enrollment, January 2017, through October 2020. Before adjusting for differences in demographics, quitline treatment engagement, and unadjusted quit rates, the quit rates for vapers were significantly higher. However, after adjusting for demographic and treatment engagement variables, 6-month quit rates among vapers did not differ significantly from rates among smokers.

**Summary**

**What is already known on this topic?**

E-cigarettes are the most commonly used tobacco product in the US after cigarettes, and the number of exclusive vapers who call quitlines is increasing. Standard quitline services are effective at helping smokers quit, but it is unclear whether these services are effective for exclusive vapers.

**What is added by this report?**

Our secondary data analysis compared cessation rates among quitline callers who smoked cigarettes only with those who vaped only and found outcomes to be similar for both groups.

**What are the implications for public health practice?**

Although this is a preliminary analysis, standard quitline protocols for smoking appear effective when applied to vaping cessation.

## Objective

Quitlines are effective in helping smokers quit, are cost-effective tobacco cessation methods, and are available free of charge in all 50 US states. They offer a range of services, including telephone counseling, print materials, cessation medications, and web- and print-based interventions (1–4). Cigarettes are the most commonly used tobacco product in the US, followed by e-cigarettes (5). Most e-cigarette users are also smokers; however, the number of quitline callers who only vape — use e-cigarettes exclusively — is increasing (6,7). The evidence base for helping smokers quit is established; however, little is known about quitting or withdrawing from e-cigarette products (3,7,8). Although exclusive vapers call quitlines for help, no existing published record shows the effectiveness of standard quitline intervention for vaping cessation. To remedy this gap in the literature, we analyzed data collected from employer-sponsored quitline participants and compared demographics, treatment engagement, and cessation outcomes for exclusive vapers with exclusive smokers to examine the effectiveness of existing quitline protocols for helping exclusive vapers quit.

## Methods

Our secondary analysis of quitline data examined participants who enrolled in employer sponsored quitlines operated by Optum, Inc (Optum) from January 2017 through October 2020. Participants were exclusive vapers or exclusive smokers at the time of program registration and had completed at least 1 coaching call. Our analysis was reviewed by the United Health Group Office of Human Research Affairs Institutional Review Board and determined exempt.

Optum’s Quit For Life quitline treatment program consists of 5 coaching calls, free nicotine replacement therapy (NRT), and access to integrated printed material, text messaging, and online cessation support. The program is available as a public health service in 23 states and to employees and members through over 1,000 employers and health plans. Its effectiveness and cost-effectiveness have been previously demonstrated for tobacco cessation (1–4,9). The program focuses on 5 keys for quitting tobacco: setting a quit date, using medications effectively, learning to cope with cravings, tobacco-proofing the smoker’s environment, and using social support (9). Some employers offer a financial incentive for completing all 5 coaching calls. Quit coaches receive ongoing training on e-cigarettes and their cessation protocol uses the 5 keys. NRT dosing for e-cigarette use is based on time to first use in the morning and is adjusted on the basis of the coach’s assessment of dependence level and the participant’s previous experiences with NRT.

We collected data on participant demographic characteristics and tobacco use at the time they registered in the program. Participant record systems captured program engagement data about coaching call completion and provision of NRT. Outcomes from 30-day point prevalence abstinence were self-reported and were collected at 6 months via a standard program evaluation survey. Participants were first invited by email and text message to log into a user portal and complete the online survey. Survey staff then attempted to contact nonrespondents by telephone for up to 11 days.

## Results

A total of 22,845 exclusive smokers and 1,194 exclusive vapers enrolled in a quitline, received quitline treatment, and were included in our sample. Exclusive vapers were more likely to be male (vapers, 60.3%; smokers, 43.2%; *P* < .001) and were younger on average (vapers: mean, 43.0, SD, 11.7; smokers: mean, 49.5, SD, 11.2; *P* < .001). Exclusive vapers also completed a higher number of coaching calls on average (vapers: mean, 3.8, SD, 1.7; smokers: 3.2, SD, 1.8; *P* < .001) and were less likely to be mailed NRT from the quitline program (vapers, 43.8%; smokers, 69.8%; *P* < .001) (Table). Exclusive smokers smoked an average of 13.8 cigarettes per day (SD, 9.2). Exclusive vapers had a higher proportion of missing data on time to first product use in the morning (vapers, 14.6% vs smokers, 4.2%); 24.2% of exclusive smokers and 20.4% of exclusive vapers reported first use within 5 minutes after waking.

**Table:** Characteristics of Callers to Employer-Sponsored Quitlines, by Tobacco Type at Time of Registration, January 2017–October 2020

Characteristic	Total	Exclusive smokers	Exclusive vapers
**Total, n (%)**	24,039	22,845 (95.0)	1,194 (5.0)
**Age group, no. (%), y**			
18–24	5,834 (24.3)	111 (0.5)	43 (3.6)
25–40	12,475 (51.9)	5,304 (23.2)	466 (39.0)
41–59	758 (3.2)	11,884 (52.0)	501 (42.0)
≥60	4,197 (17.5)	4,844 (21.2)	111 (9.3)
Missing data	775 (3.2)	702 (3.1)	73 (6.1)
Mean (SD)	49.2 (11.3)	49.5 (11.2)	43.0 (11.7)^a^
**No. (%) Male**	10,587 (44.0)	9,867 (43.2)	720 (60.3)^a^
**Cigarettes per day, mean (SD)**	—b	13.8 (9.2)	—b
**Time to first use,^c^ no. (%) min**			
≤5	5,763 (24.0)	5,519 (24.2)	244 (20.4)
6–30	8,182 (34.0)	7,918 (34.7)	264 (22.1)
31–60	3,745 (15.6)	3,587 (15.7)	158 (13.2)
>60	4,599 (19.1)	4,358 (19.1)	241 (20.2)
Does not know/refused	608 (2.5)	495 (2.2)	113 (9.5)
Missing data	1,142 (4.8)	968 (4.2)	174 (14.6)
**Received NRT sent from quitline, no. (%)**	16,460 (68.5)	15,937 (69.8)	523 (43.8)^a^
**Calls completed, mean (SD)**	3.3 (1.8)	3.2 (1.8)	3.8 (1.7)^a^
1–4	14,747 (61.3)	14,192 (62.1)	555 (46.5)
≥5	9,292 (38.7)	8,653 (37.9)	639 (53.5)

Abbreviations: NRT, nicotine replacement therapy.

a *P* <.001.

b Not relevant for exclusive e-cigarette users.

c Time to first use is the time between awaking and the first cigarette or vape used that day.

A total of 488 (40.9%) exclusive vapers and 8,382 (36.7%) exclusive smokers responded to our survey at 6 months (*P* = .004). The respondent 30-day point prevalence abstinence for exclusive vapers was 62.5% (305 of 488) and for exclusive smokers was 58.5% (4,900 of 8,382), *P* = .08. The 30-day point prevalence abstinence intent-to-treat rate, which assumes participants lost to follow-up were continued users, was 25.5% (305 of 1,194) for exclusive vapers and 21.4% (4,900 of 22,845), *P* < .001 for exclusive smokers (Figure). After adjusting for age, gender, NRT provided, and call count, the intent-to-treat rates between exclusive smokers and exclusive vapers were no longer significant. Among survey respondents, 95.3% (465 of 488) of exclusive vapers and 93.8% (7,863 of 8,382) of exclusive smokers reported they were satisfied with treatment.

**Figure:**
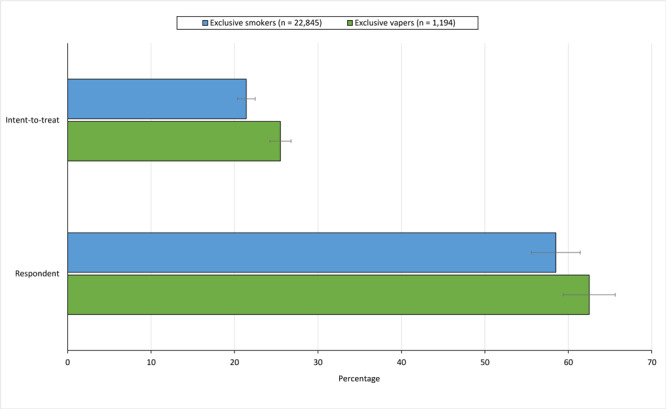
Thirty-day self-reported smoking and vaping abstinence outcomes for callers to employer-sponsored quitlines 6 months after registration. Quitline registration was completed from January 2017 through October 2020. Intent-to-treat assumes all participants who did not respond to follow-up were continued users. Error bars indicate 95% CIs.

**Table:** 

Outcome	Exclusive vapers, % (95% CI) (n = 1,194)	Exclusive smokers, % (95% CI) (n = 22,845)
Respondent quit rates	62.5 (59.4–65.6)	58.5 (55.6–61.4)
Intent-to-treat quit rates	25.5 (24.2–26.8)	21.4 (20.3–22.5)

## Discussion

Quitlines provide evidence-based, accessible, and cost-effective services for tobacco users (3,9). Our secondary data analysis did not find meaningful differences in cessation rates between exclusive smokers and exclusive vapers, suggesting that existing quitline protocols successfully support people attempting to quit vaping.

The lower number of exclusive vapers vis-à-vis exclusive smokers (1,194 vs 22,845) in our sample reflects prevalence in the adult US population, which is about 12.5% for exclusive smokers and 1% for exclusive vapers (5,10). Exclusive vapers in the sample were more likely to be younger and male on average than exclusive smokers. These differences are also consistent with the US population, where exclusive vapers are younger than exclusive smokers and men are twice as likely to vape as women (4.3% vs 2.3%), a relatively higher proportion compared with the difference between male (15.3%) and female exclusive smokers (12.7%) (10,11).

A combination of behavioral counseling and Food and Drug Administration-approved medications, such as NRT, offers the best chance of success in quitting smoking (3,9). Evidence is limited on services critical for vaping cessation. For example, although NRT was designed to address nicotine addiction and popular pod-based e-cigarettes deliver nicotine at levels similar to cigarettes, NRTs are not FDA-approved for vaping cessation. No published studies have examined NRT for exclusive vapers (12).

Although quit rates were similar between the groups, exclusive vapers engaged in more coaching calls on average than exclusive smokers but were less likely to be given NRT. Why exclusive vapers were more likely to complete calls is unclear. Possibly more exclusive smokers were motivated to enroll to receive NRT and were less interested in coaching. This could explain, in part, why NRT was more likely to be provided to exclusive smokers.

Our study had limitations. The analysis excluded users of tobacco products other than cigarettes and e-cigarettes, participants who used multiple tobacco products, and those who used both cigarettes and e-cigarettes at the time of enrollment. Because limited data were gathered in our study, we were unable to describe patterns of e-cigarette use, reasons for use, and dependence or to consider differences in other participant characteristics such as education, income, or eligibility for incentives. Finally, outcome survey response rates of approximately 40% may affect the generalizability of these findings to survey nonresponders, but findings do align with other quitline program evaluations that did not use response incentives (13).

More research is needed on vaping cessation and ways it might need to differ from approaches that help cigarette smokers quit. Our analysis provides a preliminary, but promising, look at quitline protocols for smoking applied to vaping cessation; however, additional information about needed tailoring of coaching or NRT could improve outcomes.
